# Impact of Cold Storage on Bioactive Compounds and Their Stability of 36 Organically Grown Beetroot Genotypes

**DOI:** 10.3390/foods10061281

**Published:** 2021-06-04

**Authors:** Khadijeh Yasaminshirazi, Jens Hartung, Michael Fleck, Simone Graeff-Hönninger

**Affiliations:** 1 Group of Cropping Systems and Modelling, Institute of Crop Science, University of Hohenheim, Fruwirthstr. 23, 70599 Stuttgart, Germany; simone.graeff@uni-hohenheim.de; 2 Department of Biostatistics, Institute of Crop Science, University of Hohenheim, Fruwirthstr. 23, 70599 Stuttgart, Germany; jens.hartung@uni-hohenheim.de; 3 Kultursaat e.V., Kronstraβe 24, 61209 Echzell, Germany; michael.fleck@kultursaat.org

**Keywords:** beetroot, organic farming, storage, bioactive compounds, betalain, nitrate, sugar, phenolic compounds, total dry matter

## Abstract

In order to exploit the functional properties of fresh beetroot all year round, maintaining the health-benefiting compounds is the key factor. Thirty-six beetroot genotypes were evaluated regarding their content of total dry matter, total phenolic compounds, betalain, nitrate, and total soluble sugars directly after harvest and after cold storage periods of one and four months. Samples were collected from two field experiments, which were conducted under organic conditions in Southwestern Germany in 2017 and 2018. The outcome of this study revealed a significant influence of genotype (*p* < 0.05) on all measured compounds. Furthermore, significant impacts were shown for storage period on total dry matter content, nitrate, and total phenolic compounds. The medians of nitrate content based on the genotypes studied within the experiment ranged between 4179 ± 1267–20,489 ± 2988 mg kg^−1^ DW (dry weight), and that for the total phenolic compounds varied between 201.45 ± 13.13 mg GAE 100 g^−1^ DW and 612.39 ± 40.58 mg GAE 100 g^−1^ DW (milligrams of gallic acid equivalents per 100 g of dry weight). According to the significant influence of the interactions of storage period and genotype on total soluble sugars and betalain, the decrease or increase in the content of the assessed compounds during the cold storage noted to be genotype-specific. Therefore, to benefit beetroots with retained quality for an extended time after harvest, selection of the suitable genotype based on the intended final use is recommended.

## 1. Introduction

Containing a high amount of health-promoting compounds [1], suitable cultivation and storage [2] brought attention to the use of beetroot (*Beta vulgaris* subsp. *vulgaris* L.) and its products. Classification of beetroot as a super food has increased the importance of this vegetable [3]. Beetroot is included in the ten vegetables with the highest antioxidant capacity due to its promising amount of betalain and phenolic compounds [4] as well as the presence of carotenoid and ascorbic acid [5,6].

Betalains are nitrogen-containing, water-soluble plant secondary metabolites, which are derivatives of betalamic acid [7] and can be found in different parts of plants, but are restricted to the *Caryophyllales* order [8]. Red beetroot, as one of the chief and the most commercially sources [9] of betalain, has been approved by European Union to be used as a natural colorant (E162) [8] in dairy, confectionery, beverages, and meat products [10]. Based on the nature of the substituent of betalamic acid residue, betalains can be divided into two major groups: betacyanin which is responsible for red-violet color, and betaxanthin representing the yellow-orange color [11]. Investigating different beetroot genotypes indicated that the main betacyanins in beetroot are betanin, isobetanin, betanidin, and isobetanidin, and the chief betaxanthin components are vulgaxanthin I and vulgaxanthin II [12]. In spite of the fact that betalains show low stability at higher temperatures [13], due to its high stability in the wide range of pH values (between three and seven [9]), betacyanins are considered to be a better choice providing a red-violet color range for coloring foods with a low acid content, compared to anthocyanins. Anthocyanins are the most common pigments for this color range [14], despite showing instability at pH values above three [15]. Indicating anti-lipidemic, anti-cancer, and antimicrobial activities made betalain a beneficial component for human health [16]. Moreover, flavonoids, phenolic acids, and various organic and inorganic acids, which are the major phenolic compounds of beetroot [5], further increase the anti-radical activity of this crop, which leads to preventing cancer and cardiovascular disease [17,18]. Total phenolics are part of fruits and vegetables’ bioactive compounds, which not only benefit plants with assisting the growth and protective mechanisms under abiotic stress conditions [19], but also promote human health with influencing the functional qualities of plant products [20].

In addition, the potential profiting effects of nitrate in beetroot on human health have drawn a lot of attention. Green leafy vegetables including beetroot are considered as major dietary sources of nitrate [21], which is the chief contributor in nitric oxide production [19,22]. Nitric oxide demonstrated an essential role in the gastric [23] and cardiovascular [24] regulations. Latterly, preventing ischemia-reperfusion damages, regulating the blood pressure [25], enhancing muscle efficiency and endurance [1], were reported as the potential positive effects of dietary nitrate. Therefore, beetroot has been recently used as a dietary supplement for patients with hypertension or cardiovascular diseases [23] or as powder formulation in different products, such as yogurt, drinks, and snacks, for consumption by athletes before physical exercises [5]. Moreover, the contribution of nitric oxide in the improvement of seed germination, growth performance, and mineral adjustment in plants under different abiotic stress conditions has been reported [26,27,28]. Furthermore, the sugar composition of beetroot was reported as a dominant proportion of sucrose (91.6%) [29], with a small and relatively similar proportion of glucose and fructose [5]. Information on the amount and composition of carbohydrates in vegetables is essential when considering the importance of sugar content in different controlled diets (such as for diabetic patients, athletes, and vegetarians) as well as in the food industry for optimization of processing practices.

Due to the rise in consumers’ awareness of the advantages of organic products [30], the demand for such products is steadily growing [29]. Comparing the quality of organic and conventional cultivation, previous studies reported higher contents of total phenolic compounds, betalain, and antioxidant capacities in organically grown beetroots. However, the extent of difference between the cultivation methods highly depends on the genotype [31]. Considering the functional characteristics of beetroot, the stability of its heath beneficial compounds plays an important role [32]. In order to have fresh beetroot throughout the year and be able to use them for the processed products with promising quality, preserving the nutritional properties of beetroot during storage is crucial. Previous studies reported the impact of genotypes, fertilization, and the storage environment on the storability of beetroot [2]. Nevertheless, an evaluation of various biologically active compounds considering a broad range of beetroot genotypes, which were cultivated organically was missing. Cold or refrigerated storage is one of the prevalent postharvest practices, which is applied for extending the shelf-life of vegetables. Due to their perishable nature, vegetables and fruits often need to be stored at low temperatures to minimize their physiological and chemical changes [33], and to extend their marketing after the harvest season [34]. Different studies claimed that advanced storage technologies, such as a controlled or modified atmosphere, are ideal options to preserve bioactive compounds in vegetables [2,35]. However, due to the high prices of such technologies, small-scale producers cannot afford them and cheap and locally-available technologies are demanded [36]. Therefore, using the genetic potential of forgotten varieties or breeding new and promising genotypes with high storability and evaluating their performances under organic farming conditions could be one of the most reasonable solutions.

The present study aimed to determine the impact of genotype and cold storage period on the stability of different bioactive compounds of 36 beetroot genotypes, grown under organic farming conditions in Southwestern Germany. The outcomes of this study can be beneficial for household consumers, who tend to favor fresh beetroot for extended time rather than the processed ones, for farmers to profit their grown beetroots with maintained quality and less storage loss, and for food industries accessing beetroot with retained health-promoting compounds.

## 2. Materials and Methods

### 2.1. Chemicals and Reagents

For quantification of nitrate, sulfanilamide (AppliChem GmbH, Darmstadt, Germany), ammonium chloride and hydrochloric (Th. Geyer, Renningen, Germany), sodium nitrite and ammonia solution 25% (Merck, Darmstadt, Germany), and N-(1-naphthyl)-ethylene diamine dihydrochloride (Carl Roth GmbH, Karlsruhe, Germany) were used. Regarding total phenolic content measurement, Folin–Ciocalteu reagent and gallic acid were provided by Merck (Darmstadt, Germany). Na_2_CO_3_ and methanol were purchased from AppliChem GmbH (Darmstadt, Germany) and Carl Roth GmbH (Karlsruhe, Germany), respectively. Ethanol needed for betalain analysis was purchased from Th. Geyer (Renningen, Germany).

### 2.2. Plant Materials and Sample Preparation

The beetroots analyzed in the present study were grown under organic conditions at the research station for organic farming Kleinhohenheim, University of Hohenheim, Stuttgart, Baden-Wuerttemberg, Germany (48°44′14 N, 9°12′01 E, 430 m above the sea level). Two field experiments were conducted in which, in 2017, 40 genotypes, and in 2018, 36 genotypes were cultivated. In 2017, the field experiment was conducted as row-column design with three replicates. In 2018, the experiment was carried out as non-resolvable block design, in which a block size of ten and a treatment number of 36, with six replicates for four genotypes and three replicates for 32 genotypes were applied. Although the data from all genotypes were statistically analyzed together, the results shown in the present study were limited to the 36 genotypes, which occurred in both years. Table 1 presents the detailed information on beet color, beet shape, and seed origin of the studied beetroot genotypes.

During the growth period in 2017, the mean precipitation and temperature were 77.42 mm and 17.6 °C, respectively. In 2018, the mean precipitation reached 38.2 mm and the mean temperature was 19.0 °C during the growth period. Detailed information on monthly precipitation and mean temperature, fertilization, sowing and harvest dates, and soil management practices can be found in Yasaminshirazi et al. [37].

Three randomly selected beetroots per plot were collected each year for analysis of the bioactive compounds in freshly harvested beetroots. Additionally, beetroots from each plot were stored in vegetable net sacks in a cooling chamber at 6 °C, directly after harvest. All stored beetroots met the marketability criteria (including beet diameter of 5–13 cm, no deformation or remarkable damages, diseases, etc.). Beetroots for analysis of bioactive compounds were taken from the cooling chamber one and four months after the storage. After washing and cutting the leaves-growth-base and root tail, a sectional cut of each beet (flesh including peel) was diced and mixed in order to have a homogenous sample from each plot. After collecting the diced beetroots in a plastic flask, to prevent any further enzymatic processes, samples were immediately frozen by liquid nitrogen, kept at −18 °C, and then followed by lyophilization using the Dieter Piatkowski–Forschungsgeraete freeze-dryer (Munich, Germany). The dried samples were milled using GRINDOMIX GM 200 (Retsch GmbH, Haan, Germany) up until a fine powder texture was reached. Until analysis, the powdered samples were stored in closed plastic bottles in a dark and dry box at ambient temperature. Total dry matter content (TDMC), total phenolic compounds, betalain, nitrate, and total soluble sugars of beetroots of freshly harvested samples were determined and compared with those of samples taken after one and four months of cold storage.

### 2.3. Total Dry Matter Content

Before and after freeze-drying, the diced beetroot samples (*i*) were weighed. Equation (1) was used to calculate the TDMC:

(1) $${\mathrm{TDMC}}_{i}\;[\%]=(\frac{{\mathrm{weight}\;\mathrm{after}\;\mathrm{drying}}_{i}}{{\mathrm{weight}\;\mathrm{before}\;\mathrm{drying}}_{i}})\;\text{×}\;100,$$

### 2.4. Total Phenolic Content (TPC)

The TPC quantification was conducted according to the methodology of Folin–Ciocalteu [38].

Briefly, the extraction was prepared by mixing 10 mL of methanol with approximately 0.5 g dried beetroot sample in a falcon tube. After shaking the mixture for 30 min, the tubes were placed in a centrifuge (Centrifuge 5810 R, Eppendorf AG, Hamburg, Germany) at 4000 rounds per minute (rpm) for 20 min (20 °C) for separation of supernatant from the solid phase. Later, 0.6 mL of the prepared extract was mixed with 60 mL of distilled water and 5 mL of Folin–Ciocalteu’s reagent in a 100 mL volumetric flask. After two to six minutes, with adding 25 mL of sodium carbonate (15%) and adjusting the final volume with distilled water to 100 mL, the mixture was left for two hours at room temperature. The absorbance at 760 nm was measured spectrophotometrically (Ultrospec 3100 Pro, Amersham Bioscience, Buckinghamshire, UK) and TPC was reported as mg GAE 100 g^−1^ DW. In order to draw a standard curve, six different concentrations of gallic acid solution (0.03–1.5 g L^−1^ gallic acid in distilled water) were used.

### 2.5. Betalain Content

Determination of two chief subgroups of betalains, namely betacyanin and betaxanthin, was conducted spectrophotometrically (Ultrospec 3100 Pro, Amersham Bioscience, Buckinghamshire, UK) in accordance with the method used by Koubaier et al. [39] and Sawicki et al. [40].

A mixture of roughly 0.04 g of the dried beetroot samples and 30 mL of 50% (*v*/*v*) ethanol was shaken for two hours with the speed of 100 rpm and followed by centrifuging the samples at 20 °C for 10 min with the speed of 400 rpm (Centrifuge 5810 R, Eppendorf AG, Hamburg, Germany). The absorption of betaxanthin and betacyanin was measured at 480 nm and 538 nm, respectively.

### 2.6. Nitrate Content Determination

The nitrate content was determined according to the flow injection analysis method (FIA) [21] using FIASTAR 5000 (FOSS Analytical AB, Hilleroed, Denmark). The detailed extract preparation can be found in Yasaminshirazi et al. [41].

### 2.7. Total Soluble Sugar Content

The degree of Brix corresponding to the percentage of total soluble sugar content was measured utilizing a digital handheld refractometer (Kruess, Hamburg, Germany). After measuring each sample in duplicate, their mean value was calculated directly.

### 2.8. Statistical Analysis

Data from both years and experiments were jointly analyzed using a mixed model approach. The model can be described by:

(2) $$y_{ijklmn}=\mu +\tau_{i}+\varphi_{j}+a_{k}+{\left(\tau \varphi \right)}_{ij}+{\left(\tau a\right)}_{ik}+{\left(\varphi a\right)}_{jk}+{\left(\tau \varphi a\right)}_{ijk}+b_{kl}+r_{klm}+c_{kln}+e_{ijklmn},\;$$

where $y_{ijklmn}$ is the observation of genotype *i* after storage period *j* in the *m* row, *n*th column of block *l* in year *k*, μ is the intercept, *τ_i_* is the fixed effect of genotype *i*, $\varphi_{j}$ is the fixed effect of the *j*th storage period, $a_{k}$ is the fixed effect of the *k*th year, ${\left(\tau \varphi \right)}_{ij}$, ${\left(\tau a\right)}_{ik}$, and ${\left(\tau \varphi a\right)}_{ijk}$ are the random interaction effects of the corresponding main effects, ${\left(\varphi a\right)}_{jk}$ is the fixed effect of storage period *j* and year *k*, and $b_{kl}$, $r_{klm}$, and $c_{kln}$ are the random effects of block, row and column within block. $e_{ijklmn}$ is the error of $y_{ijklmn}$. Note that the effect ${\left(\varphi a\right)}_{jk}$ is the confounded with the effect of sampling day and the interaction effect of year *k* and storage period *j*, as thus was taken as fixed in the model. Due to this confounding, the interpretation of common or marginal means should be made with caution, too. Further note that data of the same sample (beetroots form the same plot) were taken for different storage periods within one experiment. Thus, repeated data were taken on each sample. The model accounted for this repeated measures structure by allowing a first order autoregressive variance-covariance structure for random effects including the year and the residual error. Year specific variances and covariances were fitted to all random effects and the error except for year-by-genotype effects. As row and columns existed only in the first year, effects for both were fitted only for first year data. Pre-requirements of normally distributed residuals and homogeneous variance (despite the year specific variances) were checked graphically. In case of nitrate content, data were square-root transformed prior to analysis. For total phenolic compounds, betacyanin, and betaxanthin, data were logarithmically transformed prior to analysis. In both cases, means were back-transformed for presentation purpose only. Back-transformed values were denoted as medians. Standard errors were back-transformed using the delta method. In case of finding significant differences, Tukey test was used for multiple comparisons. A letter display was used to present their results [42].

Additionally, genotype-by-storage period means were calculated for all six traits. These simple means were standardized to have a mean of zero and a variance of one. A Principal Component Analysis (PCA) was applied on these standardized means. The two first components were presented via biplot using the default setting of the %biplot macro for SAS (factype = SYM). Thus, scaling of score and loading plot was done with $US^{\frac{1}{2}}$ and $VS^{\frac{1}{2}}$, respectively, where $USV^{\prime }$ is the single value decomposition of the two-dimensional approximation of the data matrix.

## 3. Results and Discussion

### 3.1. Total Dry Matter Content

The outcome of analysis of variance (ANOVA) demonstrated a significant effect of genotype on the content of total dry matter (*p* < 0.0001) (Table 2). This is in line with the findings of Kosson et al. [43] who claimed a significant influence of genotype on TDMC. Furthermore, it was revealed that the TDMC changed significantly during the cold storage (*p* = 0.0005). Likewise, significant influence of the interactions of storage period and year on the TDMC was noted (Table 2). Hagen et al. [44] stated no significant influence of cold storage for six weeks on the percentage of total dry matter in curly kale. Nonetheless, a strong influence of the storage on transpiration and consequently on the weight loss of the stored beetroot has been reported [45]. In this regard, Gawęda [45] assessed the difference of two storage methods on two beetroot cultivars and reported not only a significant difference between the two cultivars, but also a two-times-decline in dry weight of beetroots stored in polyethylene film bags than those in traditional plastic boxes, after 6 months of cold storage.

Based on the significant impacts of genotype and the interactions of storage period and year, Table 3 presents the means of TDMC of the investigated genotypes within the trial (A) and based on three storage periods in each year (B). The TDMC of the tested genotypes ranged between 11.49 ± 0.55% and 18.15 ± 0.55%. The highest contents of total dry matter were noted in the genotypes Nochowski (18.15 ± 0.55%), Chrobry (18.14 ± 0.55%), and Betina (16.94 ± 0.56%) and the lowest in the genotypes Alvro Mono (11.49 ± 0.55%), Libero RZ (12.31 ± 0.56%), and Tondo d. Ch. (13.37 ± 0.58%) (Table 3).

Considering the means of different storage periods in the first year, the TDMC directly after the harvest was 15.19 ± 0.14%. After one month of cold storage, the TDMC increased to 15.63 ± 0.14%. After a storage period of four months, the TDMC significantly decreased compared to the samples taken one month after the harvest and reached 14.95 ± 0.14%, which was not significantly different from the mean of freshly harvested beetroots. Therefore, the overall change in the TDMC was minor. In the second year, the TDMC directly after the harvest was 14.51 ± 0.21%. After one month of cold storage, the content rose significantly to 15.34 ± 0.21%. Afterwards, up to a storage period of four months, the change was not significant (Table 3). A corresponding outcome was noted by Jakopic et al. [46], who reported a higher TDMC in rutabaga turnips after a cold storage of four months. In contrast, a slight decrease in the TDMC of ten organically grown onion genotypes after five months of cold storage was noted [47]. Regarding the influence of the year, the amount of total dry matter did not differ significantly between the values of the samples from the freshly harvested beetroots in each year. Likewise, the TDMC contents after cold storage periods of one and four months did not differ significantly between both years. According to the significant increase in the TDMC within the first one month of cold storage in both years, it can be concluded that the highest amount of water loss could occur at the first four weeks of cold storage.

### 3.2. Total Phenolic Content

In accordance with the results of ANOVA, genotypes can significantly impact the amount of total phenolic compounds (*p* < 0.0001). This is in agreement with Lattanzio et al. [48], who stated the key effect of genotype on the content of phenolic contents in fresh fruit and vegetables. Furthermore, a significant influence of the storage period (*p* < 0.0001), year (*p* < 0.0001), and the interactions between storage period and year were noted (Table 2).

According to the significant impacts of genotype and the interactions of storage period and year, Table 3 exhibits the medians of TPC of the examined genotypes within the trial (A) and based on three storage periods in each year (B). The TPC in investigated red-colored beetroot genotypes varied from 322.93 ± 21.04 mg GAE 100 g^−1^ DW to 612.39 ± 40.58 mg GAE 100 g^−1^ DW measured in genotypes Robuschka and Alvro Mono, respectively. Following Alvro Mono, the cylindrical-shaped genotype Forono with 561.74 ± 37.37 mg GAE 100 g^−1^ DW and Monty RZ F1 with 519.91 ± 33.89 mg GAE 100 g^−1^ DW indicated the highest TPC. Taking all the genotypes into account, the lowest TPC possessed by the yellow-colored genotype Burpees G. (201.45 ± 13.13 mg GAE 100 g^−1^ DW), the red-white-colored Tondo d. Ch. (241.16 ± 20.35 mg GAE 100 g^−1^ DW), and white-colored Sniezna Kula (242.55 ± 16.07 mg GAE 100 g^−1^ DW). Based on the median values of different storage periods in the first year, the TPC directly after the harvest was 294.55 ± 8.57 mg GAE 100 g^−1^ DW. After one month of cold storage, a significant increase up to 341.18 ± 9.93 mg GAE 100 g^−1^ DW was noted.

Likewise, after a storage period of four months the TPC further increased and reached 437.83 ± 12.78 mg GAE 100 g^−1^ DW (Table 3). In the second year, the TPC directly after the harvest was 395.91 ± 12.59 mg GAE 100 g^−1^ DW. After one month of cold storage, it slightly decreased, however, the change was not significant. Afterwards, up to a storage period of four months, an increasing trend in the TPC was noticed (Table 3). The median values of TPC after a cold storage period of four months in both years were higher than those at harvest time, which indicated the potential of the investigated genotypes in providing a promising content of phenolic compounds for an extended time after the harvest. This is in agreement with the study of Jakopic et al. [46], who reported an increase of TPC of rutabaga root after four months of cold storage. Regarding the influence of the year, the amount of total phenolic compounds differed significantly between the values of the samples from the freshly harvested beetroots in each year. Nevertheless, the TPC after cold storage periods of one and four months did not differ significantly between both years.

Evaluation of the stability of TPC of red beetroot (var. Little Ball) during the storage in 5 °C for 196 days indicated a slight decrease in the first 63 days of storage and afterwards the change was minor [49]. In line with the results of the present study, in which both decreasing and increasing trends in the TPC during the storage period was noted, both decreases (in broccoli [50] and pomegranate [51]) and increases (in pigmented potato tuber [52]) in TPC have been reported in the previous studies. This may result from the differences between the impact’s extent of cold storage on individual constituents of phenolic compounds [49].

Corleto et al. [32] investigated the stability of TPC in beetroot juice, which were stored for 32 days at four different temperatures and significant differences were noted during the storage at refrigeration temperature (4 °C). Nevertheless, it was revealed that under refrigeration and freezing conditions, antioxidant activity and TPC remain more stable in comparison to storage at room temperature. Assessing the impact of temperature and period of storage on beetroot snack bars indicated that TPC decreased constantly during six months storage at all studied temperatures (6 °C, 22–32 °C, and 37 °C). However, the TPC loss at 6 °C was less than at higher temperatures [53]. High temperature is reported as the main factor causing the reduction of TPC in vegetables due to change in the phenolic profiles [54]. 

The cylindrical-shaped genotype, Forono, was noted to be among the genotypes with the highest TPC (Table 3), betacyanin, and betaxanthin (Table 4) after the cold storage, which can be correlated to the high antioxidant activity of this genotype. Furthermore, another cylindrical-shaped beetroot, Carillon RZ, was noted as a genotype with an average TPC and betalain content. Additionally, this genotype indicated the highest total and marketable yield as well as high resistance against the common beet disease, scab, among 15 investigated beetroot genotypes in our previous study [37] which further reveals its latent promising characteristics. In contradiction to Forono and Carillon RZ, the other studied cylindrical-shaped beetroot, Formanova, was among the red-colored genotypes with the lowest betalain content and an average TPC. This may explain the impact of genotype rather than the shape on the content of the discussed compounds.

### 3.3. Betalain Content

The findings of this study revealed a significant effect of the interaction between genotype and storage period on both betacyanin and betaxanthin content (*p* < 0.0001). Moreover, it was noted that the year impacted the betaxanthin and betacyanin contents significantly (Table 2). Kujala et al. [6] claimed that beetroot genotype, cultivation, and storage conditions can affect the content of betanin and isobetanin (the main betacyanins found in beetroot). Cejudo-Bastante et al. [55] reported the influence of storage duration and temperature on the betalain content of fruits. Generally, temperature has been reported as a key factor for the stability of the betalain [56].

Two major subgroups of betalain, betaxanthin and betacyanin, were measured in this study. Respecting the significant impact of the interactions between genotype and storage period on both betaxanthin and betacyanin contents, Table 4 demonstrates the contents based on different storage periods for each genotype separately. Considering all studied genotypes, the highest betaxanthin contents in beetroots directly after harvest were measured in the genotypes Gesche SG (6.60 ± 1.26 mg g^−1^ DW), Libero RZ (6.25 ± 1.19 mg g^−1^ DW), and Ronjana (6.16 ± 1.18 mg g^−1^ DW) and the lowest values were noted in the white-colored genotype Sniezna Kula, yellow-colored Burpees G., and red-white Tondo d. Ch., with 0.13 ± 0.03 mg g^−1^ DW, 0.20 ± 0.04 mg g^−1^ DW, and 0.22 ± 0.4 mg g^−1^ DW, respectively (Table 4). Comparing the red-colored genotypes, the betaxanthin content after one month of cold storage varied between 3.08 ± 0.59 mg g^−1^ DW and 6.73 ± 1.38 mg g^−1^ DW belonging to the genotypes Alvro Mono and Libero RZ, respectively. Following Alvro Mono, the genotypes Formanova (3.54 ± 0.69 mg g^−1^ DW), Detroit G. (4.08 ± 0.78 mg g^−1^ DW), and UB-E3 (4.21 ± 0.80 mg g^−1^ DW) possessed the lowest betaxanthin values (Table 4). However, the white-colored genotype Sniezna Kula with 0.18 ± 0.05 mg g^−1^ DW, the red-white genotype Tondo d. Ch. with 0.26 ± 0.05 mg g^−1^ DW, and yellow-colored genotype Burpees G. with 1.15 ± 0.22 mg g^−1^ DW indicated the lowest betaxanthin values when taking all studied genotypes into account (Table 4). After the storage period of four months, the betaxanthin content of the red-colored genotypes ranged between 3.05 ± 0.60 mg g^−1^ DW and 6.70 ± 1.28 mg g^−1^ DW belonging to the genotypes Alvro Mono and the cylindrical-shaped Forono, respectively. However, considering all studied genotypes, the lowest betaxanthin contents were observed in the white-colored genotype Sniezna Kula with 0.16 ± 0.05 mg g^−1^ DW, the red-white genotype Tondo d. Ch. with 0.19 ± 0.05 mg g^−1^ DW, and yellow-colored genotype Burpees G. with 1.03 ± 0.20 mg g^−1^ DW (Table 4). Regarding the influence of cold storage, in the yellow-colored genotype Burpees G., a significant increase in the betaxanthin content after one month of cold storage was observed and afterwards, up to a storage period of four months, no significant change occurred (Table 4).

The betacyanin content of the red-colored genotypes directly after the harvest ranged from 3.61 ± 0.65 mg g^−1^ DW to 8.70 ± 1.56 mg g^−1^ DW. The three highest betacyanin contents were noted in genotypes Monty RZ F1, Libero RZ, and Forono, respectively (Table 4). Taking all the examined genotypes into account, the white-colored genotype Sniezna Kula with 0.12 ± 0.03 mg g^−1^ DW, the red-white genotype Tondo d. Ch. with 0.30 ± 0.05 mg g^−1^ DW, and yellow-colored genotype Burpees G. with 0.43 ± 0.08 mg g^−1^ DW possessed the lowest betacyanin contents. After one month of cold storage, the betacyanin content of the red-colored genotypes varied between 4.13 ± 0.74 mg g^−1^ DW and 8.70 ± 1.70 mg g^−1^ DW belonging to Alvro Mono and Libero RZ, respectively, and that after a cold storage of four months, ranged between 4.28 ± 0.79 mg g^−1^ DW and 9.05 ± 1.63 mg g^−1^ DW found in the genotypes Alvro Mono and Ronjana, respectively. After cold storage periods of one and four months, the lowest betacyanin contents were measured in Burpees G., Sniezna Kula, and Tondo d. Ch., respectively (Table 4). Among all investigated genotypes, the betacyanin content in yellow-colored genotype, Burpees G., was significantly influenced by the duration of the cold storage. In this regard, a significant decrease in the betacyanin content after one month of cold storage was noted and afterwards up to a storage period of four months, no significant change was observed (Table 4).

Kujala et al. [49] studied the effect of cold storage at 5 °C on betanin and isobetanin content of the red beetroots (var. Little Ball) grown in Finland and significant differences in the amounts of betanin and isobetanin during the cold storage (0–196 days) were noted. Moreover, it was reported that the content of betanin in red beetroot peel decreased in the first 140 days of cold storage and then slightly increased. In terms of isobetanin, until 98 days, an increasing trend and afterward, up to 140 days of storage, a light decrease were noticed [49]. Maity et al. [53] investigated the effect of storage temperature and duration on betacyanin and betaxanthin contents of compressed beetroot snack bars and the maximum retention was noted in those stored at 6 °C and the content did not change significantly after four months of storage.

Moreover, storage of beetroot powder in three different temperatures (namely 10, 25, and 40 °C) indicated the minimum loss in the content of betacyanin and betaxanthin at the lowest temperature up to five weeks [56]. Yong et al. [57] investigated the effect of seven days of cold storage on betacyanin content of red pitahaya at 4 °C and reported a significant increase after six days of storage and then a slight decrease on day seven. In contrast, Obenland et al. [58] noted no significant impact of two weeks storage of red pitahaya at 5 and 10 °C on the betacyanin content.

### 3.4. Nitrate Content

The average nitrate content differed significantly between the genotypes (*p* = 0.0005) (Table 2). A corresponding result was found by Kosson et al. [43], who reported a significant influence of variety on the nitrate content in two beetroot genotypes. Moreover, a significant impact of the storage period on the nitrate content was noted (*p* < 0.0001). On the other hand, the interactions between storage period and genotype, year, and interactions between storage period and year on the nitrate content were not significant (Table 2).

Corleto et al. [32] reported a significant impact of different refrigeration temperatures and periods on the nitrate level of freshly pressed beetroot juice. Moreover, their study revealed that in the first eight days of storage at 4 °C, the nitrate value did not change significantly, while between day 8 and 32 of the storage, the nitrate level decreased drastically. In contrast, Chung et al. [59] studied the impact of cold storage on four different leafy vegetables, including spinach, crown daisy, organic Chinese spinach, and organic non-heading Chinese cabbage, and no significant changes in the nitrate content could be proven during the storage period.

**Figure 1:**
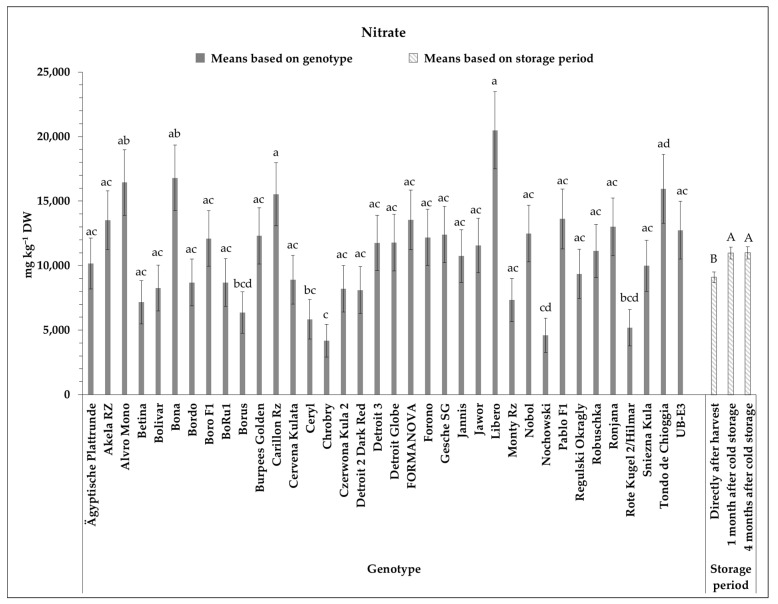
Median values of nitrate content (mg kg^−1^ DW) of 36 beetroot genotypes grown at the research station Kleinhohenheim within the years 2017 and 2018, based on genotype and storage period. Results represent the median values ± asymptotic standard error. Medians covered by at least one identical lower-case letter did not differ significantly between genotypes at experiment-wise Type 1 error α = 0.05. Medians covered by at least one identical upper-case letter did not differ significantly between storage periods at experiment-wise Type 1 error α = 0.05.

According to the significant effect of genotype and storage period on the content of nitrate, Figure 1 demonstrates the medians of nitrate content of the studied genotypes as well as medians based on three storage periods studied within the experiment. Moreover, the precise median and asymptotic standard error values of nitrate content are available in Table S1 in the supplementary material. The nitrate values exhibited in Figure 1 were reported on a dry weight basis, while nitrate content in literature is often also presented on a fresh weight (FW) basis. Consequently, to compare the findings of this work with other studies, the TDMC should be considered. The amount of nitrate of the studied genotypes ranged between 4179 ± 1267 mg kg^−1^ DW and 20,489 ± 2988 mg kg^−1^ DW found in Chrobry and Libero RZ, respectively (Figure 1). Following Libero RZ, the genotypes Bona (16,794 ± 2539 mg kg^−1^ DW) and Alvro Mono (16,438 ± 2539 mg kg^−1^ DW) indicated the highest nitrate contents within the trial. The second and third lowest nitrate content were measured in genotypes Nochowski with 4602 ± 1329 mg kg^−1^ DW, and Hilmar with 5190 ± 1411 mg kg^−1^ DW, respectively.

With regards to the medians based on the storage period, the nitrate content of 9095 ± 414 mg kg^−1^ DW was calculated for all genotypes in both years for samples collected directly after the harvest. After one month of cold storage, the median nitrate content increased significantly and reached a median of 10,977 ± 459 mg kg^−1^ DW. No further significant increase was observed after a cold storage period of four months (Figure 1). Consequently, based on the outcome of this study, the main change in the nitrate content of the investigated genotypes arose within the first four weeks of storage.

According to the point that the nitrate content may not only vary between the genotypes but also among the cultivars of the same species and plant tissues [60,61,62], assessing a greater number of plants per genotype for having a better evaluation can be recommended.

### 3.5. Total Soluble Sugar Content

According to the statistical analysis, interactions between storage period and genotype were significant (*p* = 0.0121). Viskelis et al. [2] reported no significant change of total sugar content of 11 beetroot genotypes, grown in Lithuania, during the storage at 1 ± 1 °C and relative humidity of 90–95% for the storage period of seven months. Nonetheless, in the case of four common genotypes in both studies, namely Bona, Boro F1, Detroit 2 D. R., and Pablo F1, despite the lower values in our study, the storage period did not impact the content of total soluble sugars significantly. Thus, it disclosed that the influence of cold storage might be genotype-dependent.

To better appraise the impact of cold storage on sugar content, it is noteworthy to know the amount of sugar in freshly harvest beetroot. In this regards, the freshly harvested beetroots in this study contained the total soluble sugar content in the range of 8.55 ± 0.67 °Bx to 15.43 ± 0.67 °Bx possessing by the genotypes Alvro Mono and Nochowski, respectively. After one month of cold storage, the three highest total soluble sugar contents belonged to the genotypes Nochowski, Chrobry, and Cervena K., with 14.88 ± 0.67 °Bx, 13.96 ± 0.67 °Bx, and 13.89 ± 0.67 °Bx, respectively (Table 5). The genotypes Alvro Mono (9.00 ± 0.46 °Bx), Bolivar (9.64 ± 0.48 °Bx), and Libero RZ (9.64 ± 0.67 °Bx) exhibited the lowest total soluble sugar contents. After a cold storage period of four months, the total soluble sugar content ranged between 8.41 ± 0.71 °Bx and 14.92 ± 0.67 °Bx, belonging to the genotypes Alvro Mono and Chrobry, respectively. Following the genotype Alvro Mono, the red-colored Libero RZ, red-white-colored Tondo d. Ch., and yellow-colored Burpees G. indicated the lowest total soluble sugar contents.

Depending on the stored crop, the effect of cold storage on the total soluble sugar content can be different. Hagen et al. [63] stated a significant decrease in the total content of soluble sugars in curly kale stored for six weeks at 1 °C, while an increase in sugar content was noted in potato tubers stored for six months at 2–4 °C. Jakopic et al. [46] investigated the change in the content of soluble sugars in rutabaga during the cold storage and an increase in the first month of storage and afterwards up to the cold storage for four months a slight decrease was reported. Barboni et al. [64] investigated the impact of cold storage on the total soluble sugar content of kiwi fruit and an increase in the first seven weeks and a constant amount after 21 weeks of the storage was noted. This reveals the considerable variation in the effect of cold storage on vegetables and fruits, which is highly dependent on the species [65].

### 3.6. Principal Component Analysis

**Figure 2:**
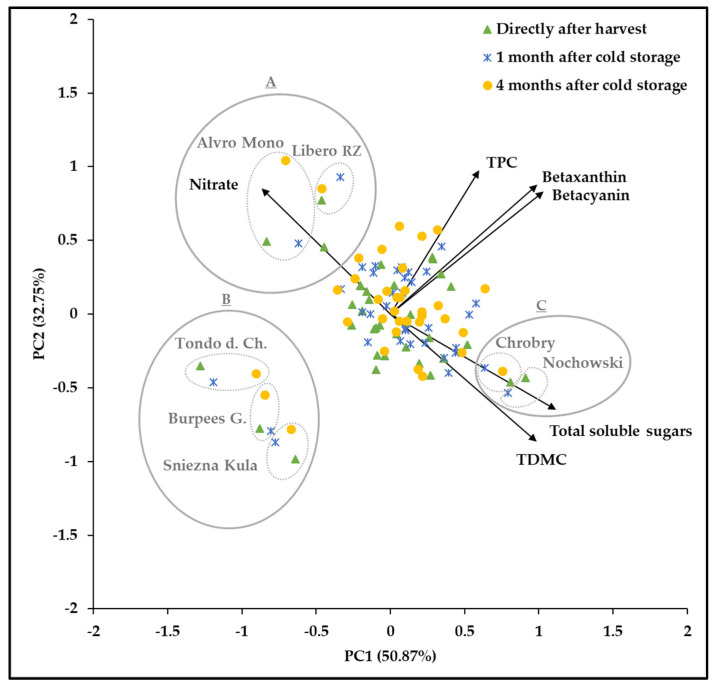
Biplot of the impact of the interactions of genotype-by-storage period means on the traits TDMC, TPC, betaxanthin, betacyanin, nitrate, and total soluble sugars for 36 beetroot genotypes grown at the research station Kleinhohenheim within the years 2017 and 2018, directly after harvest, and after the cold storage periods of one and four months. Group A shows the genotypes with the highest nitrate contents, group B the genotypes with the highest total soluble sugar contents, and group C, the non-red-colored genotypes. PC1 and PC2 are principal components 1 and 2.

The biplot of the genotype-by-storage period means (Figure 2) demonstrated that more than 83% of the variation is elucidated by two examined components.

It revealed that the TDMC and total soluble sugar content are highly positively correlated, whereas, high negative correlations between these two traits and nitrate content was noted. This is in agreement with Anjana and Iqbal [66], who reported a negative correlation between the sugar and nitrate contents and a positive correlation between carbohydrate concentration and TDMC. The biplot further confirmed that the genotypes Alvro Mono and Libero RZ, which indicated the highest nitrate values possessed the lowest sugar content among the studied genotypes. On the other hand, the highest total soluble sugar contents were noted in genotypes Chrobry and Nochowski, which were included in the genotypes with the lowest nitrate content. Moreover, the biplot visualized a high positive correlation between the contents of betacyanin and betaxanthin. Likewise, the contents of these two compounds were positively correlated with the TPC. Corresponding findings were reported by Kugler et al. [67] regarding a positive correlation between betacyanin and betaxanthin, by Kujala et al. [49] about a positive correlation between betacyanin and TPC, and Čanadanović- Brunet et al. [68] stated a significant correlation between the total phenolic compounds and betaxanthin. The biplot of the interactions of genotype and storage period approved that the lowest TPC and betalain content belonged to the non-red genotypes, including Tondo d. Ch., Burpees G., and Sniezna Kula.

## 4. Conclusions

Besides investigating a great number of beetroot genotypes and disclosing a high genetic variability regarding the content of the bioactive compounds, this study examined the storability of beetroot genotypes at a low temperature and the extent of change in their compositional quality in order to prolong the use of this vegetable for an extended time after the harvest. However, to have a thorough insight on the genetic potential of the examined beetroot genotypes for their application in various sections, the agronomic performance, and their sensory quality should be additionally considered. The genotype ‘Chrobry’ was characterized by the lowest nitrate content and indicated a high total soluble sugar content with no significant changes during four months of cold storage, thus, it may be of interest for beetroot juice production. ‘Nochowski’ and ‘Cervena K.’ which were among the top three genotypes with the highest total soluble sugar contents directly after the harvest and retained contents after one month of cold storage, indicated a significant decrease in the amount of total soluble sugars after four months of cold storage. Therefore, it is beneficial to use these beetroot genotypes freshly or within the first month of storage, when a high sugar content is desired. The genotypes ‘Forono’, ‘Ronjana’, ‘Monty RZ F1′, and ‘Nobol’ which were characterized by a high amount of betacyanin and betaxanthin, and their stability during a cold storage period of four months, can be of interest for the use as natural food colorants. The cylindrical-shaped genotype ‘Forono’ characterized by a high content of betalain and TPC, can serve as an option for value-added food products. Further studies considering beet firmness, and impact of other atmospheric conditions such as relative humidity to further improve the storability of beetroot, can be recommended.

## Figures and Tables

**Table 1:** List of the 36 investigated beetroot genotypes indicating the beet color, shape, and seed origin.

Genotype	Beet Color	Shape	Seed Origin
Ägyptische Plattrunde (Ä. P.)	red	flat-spherical	Sativa (DE)
Akela Rijk Zwaan (RZ)	red	spherical	Rijk Zwaan (NL)
Alvro Mono	red	spherical	Vitalis (US)
Betina	red	spherical	Moravo Seeds (CZ)
Bolivar	red	spherical	Hild (DE)
Bona	red	spherical	Moravo Seeds (CZ)
Bordo	red	spherical	Seklos (LT)
Boro F1	red	spherical	Bejo (DE)
BoRu1	red	spherical	Kultursaat e.V. (DE)
Borus	red	spherical	Spójnia (PL)
Burpees Golden (Burpees G.)	yellow	spherical	Bingenheimer S. AG (DE)
Carillon RZ	red	cylindrical	Rijk Zwaan (NL)
Cervena Kulata (Cervena K.)	red	spherical	Moravo Seeds (CZ)
Ceryl	red	spherical	Spójnia (PL)
Chrobry	red	spherical	Spójnia (PL)
Czerwona Kula 2 (Czerwona K. 2)	red	spherical	Spójnia (PL)
Detroit 2 Dark Red (Detroit 2 D. R.)	red	spherical	Samen Schenker (DE)
Detroit 3	red	spherical	Caillard (FR)
Detroit Globe (Detroit G.)	red	spherical	King Seed (UK)
Formanova	red	cylindrical	Sativa (DE)
Forono	red	cylindrical	Bingenheimer S. AG (DE)
Gesche SG	red	spherical	Christiansens Biolandhof (DE)
Jannis	red	spherical	Bingenheimer S. AG (DE)
Jawor	red	spherical	Snówidza (PL)
Libero RZ	red	spherical	Rijk Zwaan (NL)
Monty RZ F1	red	spherical	Rijk Zwaan (NL)
Nobol	red	spherical	Vilmorin (PL)
Nochowski	red	spherical	Spójnia (PL)
Pablo F1	red	spherical	Bejo (DE)
Regulski Okragly (Regulski O.)	red	spherical	Pnos (PL)
Robuschka	red	spherical	Bingenheimer S. AG (DE)
Ronjana	red	spherical	Bingenheimer S. AG (DE)
Hilmar	red	spherical	Hild (DE)
Sniezna Kula	white	spherical	Torseed (PL)
Tondo de Chioggia (Tondo d. Ch.)	red-white	spherical	Bingenheimer S. AG (DE)
UB-E3	red	spherical	U.Behrendt (DE)

**Table 2:** ANOVA of results of total dry matter, total phenolic compounds, betaxanthin, betacyanin, nitrate, and total soluble sugars of 36 beetroot genotypes grown in research station Kleinhohenheim within the years 2017 and 2018 for three storage periods (storage period expressed as cold storage durations of zero (directly after harvest), one, and four months).

Effect	Total Dry Matter Content	Total Phenolic Compounds	Betaxanthin	Betacyanin	Nitrate	Total Soluble Sugars
Genotype	<0.0001	<0.0001	<0.0001	<0.0001	0.0005	<0.0001
Storage period	0.0005	<0.0001	<0.0001	<0.0001	<0.0001	n.s. ^1^
Genotype × Storage period	n.s.	n.s.	<0.0001	<0.0001	n.s.	0.0121
Year	n.s.	<0.0001	0.0001	<0.0001	n.s.	0.0008
Storage period × Year	0.0009	0.0001	n.s.	n.s.	n.s.	<0.0001

^1^ not significant.

**Table 3:** Mean and median values of total dry matter content (%) and total phenolic content (mg GAE 100 g^−1^ DW) of 36 beetroot genotypes grown at the research station Kleinhohenheim within the years 2017 and 2018. Results represent the mean (median) values ± (approximate) standard error. In section (A), means (medians) followed by at least one identical lower-case letter in one column did not differ significantly between genotypes at experiment-wise Type 1 error α = 0.05. In section (B), means (medians) followed by at least one identical lower-case letter in one column did not differ significantly within different storage periods at experiment-wise Type 1 error α = 0.05 and means (medians) followed by at least one identical upper-case letter in one column did not differ significantly within year at experiment-wise Type 1 error α = 0.05.

		(A) Means (Medians) Based on Genotype	
Genotype		TDMC (%)	Total Phenolic Content (mg GAE 100 g^−1^ DW)
Ä. P.		14.77 ^bcde^ ± 0.55	417.20 ^ac^ ± 27.18
Akela RZ		14.81 ^bcde^ ± 0.55	413.30 ^ac^ ± 26.94
Alvro Mono		11.49 ^e^ ± 0.55	612.39 ^a^ ± 40.58
Betina		16.94 ^ac^ ± 0.56	413.71 ^ac^ ± 27.41
Bolivar		14.85 ^ad^ ± 0.55	364.91 ^cdef^ ± 23.79
Bona		14.78 ^bcde^ ± 0.55	405.26 ^ac^ ± 26.41
Bordo		16.79 ^ac^ ± 0.55	449.00 ^ac^ ± 29.26
Boro F1		13.84 ^bcde^ ± 0.55	414.61 ^ac^ ± 27.03
BoRu1		14.85 ^ad^ ± 0.55	417.89 ^ac^ ± 27.25
Borus		16.39 ^ab^ ± 0.56	376.35 ^bcde^ ± 25.94
Burpees G.		14.87 ^ad^ ± 0.55	201.45 ^i^ ± 13.13
Carillon RZ		13.81 ^bcde^ ± 0.55	432.35 ^ac^ ± 28.19
Cervena K.		16.15 ^ab^ ± 0.56	465.96 ^ac^ ± 31.57
Ceryl		16.25 ^ab^ ± 0.55	426.45 ^ac^ ± 27.80
Chrobry		18.14 ^a^ ± 0.55	449.16 ^ac^ ± 29.26
Czerwona K. 2		15.92 ^ab^ ± 0.55	355.25 ^cdefg^ ± 24.04
Detroit 2 D. R.		15.64 ^ad^ ± 0.56	418.26 ^ac^ ± 28.83
Detroit 3		14.30 ^bcde^ ± 0.55	403.59 ^ac^ ± 26.73
Detroit G.		14.89 ^ad^ ± 0.56	350.31 ^cdefg^ ± 24.15
Formanova		14.46 ^bcde^ ± 0.55	397.19 ^bcd^ ± 26.31
Forono		15.21 ^ad^ ± 0.55	561.74 ^ab^ ± 37.37
Gesche SG		15.25 ^ad^ ± 0.55	484.20 ^ac^ ± 31.56
Jannis		14.68 ^bcde^ ± 0.55	414.71 ^ac^ ± 27.02
Jawor		15.17 ^ad^ ± 0.55	430.75 ^ac^ ± 28.07
Libero RZ		12.31 ^de^ ± 0.56	408.49 ^ac^ ± 29.38
Monty RZ F1		15.65 ^ad^ ± 0.55	519.91 ^ad^ ± 33.89
Nobol		14.20 ^bcde^ ± 0.55	456.04 ^ac^ ± 29.74
Nochowski		18.15 ^a^ ± 0.55	486.33 ^ac^ ± 31.69
Pablo F1		14.58 ^bcde^ ± 0.55	440.53 ^ac^ ± 29.33
Regulski O.		15.89 ^ab^ ± 0.55	381.10 ^bcde^ ± 24.84
Robuschka		15.57 ^ad^ ± 0.55	322.93 ^cefgh^ ± 21.04
Ronjana		15.39 ^ad^ ± 0.55	508.49 ^ad^ ± 33.16
Hilmar		15.01 ^ad^ ± 0.55	420.64 ^ac^ ± 27.41
Sniezna Kula		15.65 ^ad^ ± 0.55	242.55 ^fi^ ± 16.07
Tondo d. Ch.		13.37 ^bde^ ± 0.58	241.16 ^ei^ ± 20.35
UB-E3		16.06 ^ab^ ± 0.55	393.07 ^bcd^ ± 26.04
		**(B) Means (Medians) Based on Storage Period**	
	**Storage Period**		
Year 1	Directly after harvest	15.19 ^abA^ ± 0.14	294.55 ^cB^ ± 8.57
	1 month after cold storage	15.63 ^aA^ ± 0.14	341.18 ^bA^ ± 9.93
	4 months after cold storage	14.95 ^bA^ ± 0.14	437.83 ^aA^ ± 12.78
Year 2	Directly after harvest	14.51 ^bA^ ± 0.21	395.91 ^aA^ ± 12.59
	1 month after cold storage	15.34 ^aA^ ± 0.21	379.09 ^aA^ ± 12.17
	4 months after cold storage	15.46 ^aA^ ± 0.22	425.18 ^aA^ ± 14.47

**Table 4:** Median values of betaxanthin (mg g^−1^ DW) and betacyanin (mg g^−1^ DW) of 36 beetroot genotypes grown at the research station Kleinhohenheim within the years 2017 and 2018, directly after harvest, and after the cold storage periods of one and four months. Results represent the median values ± approximate standard error. Medians followed by at least one identical lower-case letter in one column did not differ significantly between genotypes at experiment-wise Type 1 error α = 0.05. Medians followed by at least one identical upper-case letter in one row did not differ significantly between storage periods at experiment-wise Type 1 error α = 0.05.

Genotype	Betaxanthin (mg g^−1^ DW)			Betacyanin (mg g^−1^ DW)		
	Directly after Harvest	1 Month after Cold Storage	4 Months after Cold Storage	Directly after Harvest	1 Month after Cold Storage	4 Months after Cold Storage
Ä. P.	4.41 ^aA^ ± 0.84	4.62 ^abA^ ± 0.88	4.40 ^abA^ ± 0.84	6.12 ^abcA^ ± 1.10	6.17 ^aA^ ± 1.11	5.42 ^aA^ ± 0.97
Akela RZ	4.44 ^aA^ ± 0.85	5.70 ^aA^ ± 1.09	5.04 ^abA^ ± 0.96	6.65 ^abA^ ± 1.19	8.02 ^aA^ ± 1.44	6.77 ^aA^ ± 1.22
Alvro Mono	2.69 ^aA^ ± 0.51	3.08 ^adA^ ± 0.59	3.05 ^bcdA^ ± 0.60	3.61 ^abcdA^ ± 0.65	4.13 ^aA^ ± 0.74	4.28 ^aA^ ± 0.79
Betina	4.31 ^aA^ ± 0.82	4.73 ^abA^ ± 0.90	5.13 ^abA^ ± 1.00	5.47 ^abcdA^ ± 0.98	7.20 ^aA^ ± 1.29	6.83 ^aA^ ± 1.26
Bolivar	4.65 ^aA^ ± 0.89	4.55 ^abA^ ± 0.87	5.28 ^abA^ ± 1.01	6.30 ^abcA^ ± 1.13	5.93 ^aA^ ± 1.07	6.83 ^aA^ ± 1.23
Bona	4.07 ^aA^ ± 0.78	4.76 ^abA^ ± 0.91	3.71 ^beA^ ± 0.71	5.79 ^abcA^ ± 1.04	6.39 ^aA^ ± 1.15	4.94 ^aA^ ± 0.89
Bordo	5.03 ^aA^ ± 0.96	4.48 ^abA^ ± 0.86	5.23 ^abA^ ± 1.00	6.99 ^abA^ ± 1.25	7.05 ^aA^ ± 1.27	7.28 ^aA^ ± 1.31
Boro F1	4.75 ^aA^ ± 0.91	4.88 ^aA^ ± 0.93	4.58 ^abA^ ± 0.87	6.50 ^abA^ ± 1.17	6.17 ^aA^ ± 1.11	5.74 ^aA^ ± 1.03
BoRu1	5.38 ^aA^ ± 1.03	5.88 ^aA^ ± 1.12	4.99 ^abA^ ± 0.95	7.23 ^aA^ ± 1.30	7.56 ^aA^ ± 1.36	6.34 ^aA^ ± 1.14
Borus	4.10 ^aA^ ± 0.78	4.63 ^abA^ ± 0.88	4.28 ^bcA^ ± 0.88	5.75 ^abcA^ ± 1.03	6.40 ^aA^ ± 1.15	5.63 ^aA^ ± 1.10
Burpees G.	0.20 ^bB^ ± 0.04	1.15 ^dA^ ± 0.22	1.03 ^deA^ ± 0.20	0.43 ^egA^ ± 0.08	0.09 ^bB^ ± 0.02	0.11 ^bB^ ± 0.02
Carillon RZ	4.83 ^aA^ ± 0.92	5.17 ^aA^ ± 0.99	4.72 ^abA^ ± 0.90	6.65 ^abA^ ± 1.20	7.47 ^aA^ ± 1.34	6.51 ^aA^ ± 1.17
Cervena K.	4.11 ^aA^ ± 0.79	4.49 ^abA^ ± 0.86	4.54 ^abA^ ± 0.90	5.75 ^abcA^ ± 1.03	6.30 ^aA^ ± 1.13	5.93 ^aA^ ± 1.11
Ceryl	5.57 ^aA^ ± 1.06	6.40 ^aA^ ± 1.22	5.48 ^abA^ ± 1.05	7.91 ^aA^ ± 1.42	8.55 ^aA^ ± 1.54	7.22 ^aA^ ± 1.30
Chrobry	5.93 ^aA^ ± 1.13	4.58 ^abA^ ± 0.88	4.71 ^abA^ ± 0.90	7.84 ^aA^ ± 1.41	7.69 ^aA^ ± 1.38	7.73 ^aA^ ± 1.39
Czerwona K. 2	4.51 ^aA^ ± 0.88	4.41 ^abcA^ ± 0.84	3.91 ^bcA^ ± 0.76	6.67 ^abA^ ± 1.22	6.17 ^aA^ ± 1.11	5.48 ^aA^ ± 1.01
Detroit 2 D. R.	4.67 ^aA^ ± 0.89	4.40 ^abcA^ ± 0.84	4.63 ^abA^ ± 0.95	6.65 ^abA^ ± 1.19	6.22 ^aA^ ± 1.12	6.20 ^aA^ ± 1.21
Detroit 3	5.46 ^aA^ ± 1.04	4.58 ^abA^ ± 0.87	5.41 ^abA^ ± 1.05	7.73 ^aA^ ± 1.39	6.15 ^aA^ ± 1.10	6.72 ^aA^ ± 1.24
Detroit G.	4.67 ^aA^ ± 0.89	4.08 ^adA^ ± 0.78	4.45 ^bcA^ ± 0.90	7.02 ^abA^ ± 1.26	5.59 ^aA^ ± 1.00	5.63 ^aA^ ± 1.10
Formanova	3.63 ^aA^ ± 0.69	3.54 ^adA^ ± 0.69	3.28 ^bcdA^± 0.63	5.01 ^abcdA^ ± 0.90	5.08 ^aA^ ± 0.94	5.00 ^aA^ ± 0.90
Forono	5.76 ^aA^ ± 1.10	6.39 ^aA^ ± 1.25	6.70 ^bA^ ± 1.28	8.39 ^aA^ ± 1.51	7.99 ^aA^ ± 1.46	8.97 ^aA^ ± 1.61
Gesche SG	6.60 ^aA^ ± 1.26	5.74 ^aA^ ± 1.10	5.38 ^abA^ ± 1.03	7.79 ^aA^ ± 1.40	7.73 ^aA^ ± 1.42	7.11 ^aA^ ± 1.28
Jannis	4.17 ^aA^ ± 0.80	4.81 ^abA^ ± 0.92	4.90 ^abA^ ± 0.94	5.85 ^abcA^ ± 1.05	6.49 ^aA^ ± 1.16	6.17 ^aA^ ± 1.11
Jawor	4.24 ^aA^ ± 0.81	4.23 ^adA^ ± 0.81	3.85 ^bcA^ ± 0.74	5.86 ^abcA^ ± 1.05	6.18 ^aA^ ± 1.11	5.84 ^aA^± 1.05
Libero RZ	6.25 ^aA^ ± 1.19	6.73 ^aA^ ± 1.38	5.24 ^abA^ ± 1.07	8.63 ^aA^ ± 1.55	8.70 ^aA^ ± 1.70	6.51 ^aA^ ± 1.27
Monty RZ F1	5.87 ^aA^ ± 1.12	5.40 ^aA^ ± 1.03	5.29 ^abA^ ± 1.01	8.70 ^aA^ ± 1.56	8.20 ^aA^ ± 1.47	7.32 ^aA^ ± 1.32
Nobol	5.72 ^aA^ ± 1.09	5.38 ^aA^ ± 1.03	5.83 ^abA^ ± 1.11	8.18 ^aA^ ± 1.47	7.64 ^aA^ ± 1.37	8.16 ^aA^ ± 1.47
Nochowski	6.08 ^aA^ ± 1.16	5.05 ^aA^ ± 0.97	5.95 ^abA^ ± 1.14	7.77 ^aA^ ± 1.39	7.57 ^aA^ ± 1.36	8.49 ^aA^ ± 1.53
Pablo F1	4.35 ^aA^ ± 0.83	5.00 ^aA^ ± 0.96	4.72 ^abA^ ± 0.92	6.13 ^abcA^ ± 1.10	6.36 ^aA^ ± 1.14	5.71 ^aA^ ± 1.04
Regulski O.	3.91 ^aA^ ± 0.75	4.96 ^aA^ ± 0.95	4.48 ^abA^ ± 0.86	5.93 ^abcA^ ± 1.06	6.88 ^aA^ ± 1.24	6.23 ^aA^ ± 1.12
Robuschka	3.85 ^aA^ ± 0.74	4.60 ^abA^ ± 0.88	5.34 ^abA^ ± 1.02	5.50 ^abcA^ ± 0.99	6.22 ^aA^ ± 1.12	6.70 ^aA^ ± 1.20
Ronjana	6.16 ^aA^ ± 1.18	4.68 ^abA^ ± 0.89	6.08 ^abA^ ± 1.16	8.07 ^aA^ ± 1.45	7.42 ^aA^ ± 1.33	9.05 ^aA^ ± 1.63
Hilmar	4.42 ^aA^ ± 0.84	4.73 ^abA^ ± 0.90	5.05 ^abA^ ± 0.97	5.89 ^abcA^ ± 1.06	6.54 ^aA^ ± 1.17	6.63 ^aA^ ± 1.19
Sniezna Kula	0.13 ^bA^ ± 0.03	0.18 ^eA^ ± 0.05	0.16 ^gA^ ± 0.05	0.12 ^gA^ ± 0.03	0.17 ^bA^ ± 0.04	0.15 ^bA^ ± 0.04
Tondo d. Ch.	0.22 ^bA^ ± 0.04	0.26 ^eA^ ± 0.05	0.19 ^fgA^ ± 0.05	0.30 ^fgA^ ± 0.05	0.25 ^bA^ ± 0.05	0.20 ^bA^ ± 0.05
UB-E3	3.40 ^aA^ ± 0.65	4.21 ^adA^ ± 0.80	3.50 ^bcdA^ ± 0.68	5.31 ^abcdA^ ± 0.95	5.75 ^aA^ ± 1.03	4.29 ^aA^ ± 0.79

**Table 5:** Mean values of total soluble sugars (°Bx) of 36 beetroot genotypes grown at the research station Kleinhohenheim within the years 2017 and 2018, directly after harvest, and after the cold storage periods of one and four months. Results represent the mean values ± standard error. Means followed by at least one identical lower-case letter in one column did not differ significantly between genotypes at experiment-wise Type 1 error α = 0.05. Means followed by at least one identical upper-case letter in one row did not differ significantly between storage periods at experiment-wise Type 1 error α = 0.05.

Genotype	Total Soluble Sugars (°Bx)		
	Directly after Harvest	1 Month after Cold Storage	4 Months after Cold Storage
Ä. P.	11.08 ^adA^ ± 0.67	11.77 ^adA^ ± 0.67	12.31 ^bcA^ ± 0.67
Akela RZ	11.26 ^adA^ ± 0.67	11.58 ^adA^ ± 0.67	12.10 ^bcA^ ± 0.67
Alvro Mono	8.55 ^dA^ ± 0.67	9.49 ^dA^ ± 0.67	8.41 ^cA^ ± 0.71
Betina	13.40 ^abA^ ± 0.67	13.55 ^adA^ ± 0.67	13.74 ^abA^ ± 0.71
Bolivar	11.14 ^adA^ ± 0.67	9.53 ^cdA^ ± 0.67	12.37 ^bcA^ ± 0.67
Bona	11.18 ^adA^ ± 0.67	11.14 ^adA^ ± 0.67	10.88 ^bcA^ ± 0.67
Bordo	12.04 ^adA^ ± 0.67	13.50 ^adA^ ± 0.67	12.47 ^bcA^ ± 0.67
Boro F1	10.99 ^bcdA^ ± 0.67	11.13 ^adA^ ± 0.67	11.16 ^bcA^ ± 0.67
BoRu1	11.79 ^adA^ ± 0.67	10.60 ^adA^ ± 0.67	11.53 ^bcA^ ± 0.67
Borus	12.40 ^adA^ ± 0.67	13.53 ^adA^ ± 0.67	13.38 ^abA^ ± 0.76
Burpees G.	10.66 ^bcdA^ ± 0.67	10.59 ^adA^ ± 0.67	10.29 ^acA^ ± 0.67
Carillon RZ	9.76 ^bdA^ ± 0.67	11.87 ^adA^ ± 0.67	11.15 ^bcA^ ± 0.67
Cervena K.	13.85 ^abA^ ± 0.67	13.89 ^abcA^ ± 0.67	11.88 ^bcA^ ± 0.77
Ceryl	13.47 ^abA^ ± 0.67	12.97 ^adA^ ± 0.67	13.70 ^abA^ ± 0.71
Chrobry	14.40 ^acA^ ± 0.67	13.96 ^abA^ ± 0.67	14.92 ^bA^ ± 0.67
Czerwona K. 2	12.21 ^adA^ ± 0.67	12.14 ^adA^ ± 0.67	13.44 ^abA^ ± 0.71
Detroit 2 D. R.	12.28 ^adA^ ± 0.67	12.91 ^adA^ ± 0.67	11.29 ^bcA^ ± 0.84
Detroit 3	10.98 ^bcdA^ ± 0.67	11.34 ^adA^ ± 0.67	11.22 ^bcA^ ± 0.71
Detroit G.	10.53 ^bcdA^ ± 0.67	11.47 ^adA^ ± 0.67	11.72 ^bcA^ ± 0.76
Formanova	11.15 ^adA^ ± 0.67	11.11 ^adjA^ ± 0.67	11.34 ^bcA^ ± 0.67
Forono	11.75 ^adA^ ± 0.67	12.05 ^adA^ ± 0.67	11.87 ^bcA^ ± 0.67
Gesche SG	13.07 ^abeA^ ± 0.67	12.63 ^adA^ ± 0.67	11.80 ^bcA^ ± 0.67
Jannis	11.19 ^adA^ ± 0.67	11.72 ^adA^ ± 0.67	11.87 ^bcA^ ± 0.67
Jawor	11.71 ^adA^ ± 0.67	12.22 ^adA^ ± 0.67	12.93 ^abA^ ± 0.67
Libero RZ	8.97 ^deA^ ± 0.67	9.64 ^bdA^ ± 0.67	9.60 ^acA^ ± 0.76
Monty RZ F1	11.36 ^adA^ ± 0.67	13.17 ^adA^ ± 0.67	12.90 ^bcA^ ± 0.71
Nobol	10.61 ^bcdA^ ± 0.67	11.53 ^adA^ ± 0.67	11.17 ^bcA^ ± 0.67
Nochowski	15.43 ^aA^ ± 0.67	14.88 ^aA^ ± 0.67	12.20 ^bcA^ ± 0.67
Pablo F1	10.81 ^bcdA^ ± 0.67	11.11 ^adA^ ± 0.67	11.61 ^bcA^ ± 0.81
Regulski O.	11.76 ^adA^ ± 0.67	12.26 ^adA^ ± 0.67	11.99 ^bcA^ ± 0.67
Robuschka	11.86 ^adA^ ± 0.67	12.82 ^adA^ ± 0.67	11.99 ^bcA^ ± 0.67
Ronjana	12.71 ^adA^ ± 0.67	11.71 ^adA^ ± 0.67	11.73 ^bcA^ ± 0.67
Hilmar	11.43 ^adA^ ± 0.67	12.71 ^adA^ ± 0.67	11.75 ^bcA^ ± 0.67
Sniezna Kula	11.60 ^adA^ ± 0.67	10.74 ^adA^ ± 0.67	11.50 ^bcA^ ± 0.71
Tondo d. Ch.	8.77 ^deA^ ± 0.68	9.71 ^bdA^ ± 0.71	10.07 ^bcA^ ± 0.84
UB-E3	12.70 ^adA^ ± 0.67	12.58 ^adA^ ± 0.67	13.00 ^abA^ ± 0.67

## Data Availability

The datasets presented in this study are available on request to the corresponding author.

## Supplementary Materials

The following is available online at https://www.mdpi.com/article/10.3390/foods10061281/s1, Table S1: Median values of nitrate content (mg kg^−1^ DW) of 36 beetroot genotypes grown at the research station Kleinhohenheim within the years 2017 and 2018, based on genotype and storage period.
