# Supplementary material for: Impact of Cold Storage on Bioactive Compounds and Their Stability of 36 Organically Grown Beetroot Genotypes
Source: Foods. 2021 Jun 4;10(6):1281. doi: 10.3390/foods10061281 (PMC8230214; doi:10.3390/foods10061281)
Supplement: Supplementary file 1 [file foods-10-01281-s001.zip › foods-1237524-supplementary.pdf]

Table S1. Median values of nitrate content (mg kg^{-1} DW) of 36 beetroot genotypes grown at the research station Kleinhohenheim within the year 2017 and 2018, based on genotype and storage period. Results represent the median values ± asymptotic standard error. Medians followed by at least one identical lower-case letter did not differ significantly between genotypes at experiment-wise Type 1 error $\alpha = 0.05$. Medians followed by at least one identical upper-case letter did not differ significantly between storage periods at experiment-wise Type 1 error $\alpha = 0.05$.

**(A) Medians based on genotype**	
**Genotype**	**Nitrate (mg kg^{-1} DW)**
Ä. P.	10160 ^{ac} ± 1975
Akela RZ	13509 ^{ac} ± 2279
Alvro Mono	16438 ^{ab} ± 2539
Betina	7166 ^{ac} ± 1676
Bolivar	8253 ^{ac} ± 1781
Bona	16794 ^{ab} ± 2540
Bordo	8691 ^{ac} ± 1827
Boro F1	12100 ^{ac} ± 2157
BoRu1	8685 ^{ac} ± 1848
Borus	6365 ^{bcd} ± 1613
Burpees G.	12306 ^{ac} ± 2175
Carillon RZ	15532 ^{ad} ± 2443
Cervena K.	8896 ^{ac} ± 1893
Ceryl	5838 ^{bcd} ± 1534
Chrobry	4179 ^{c} ± 1267
Czerwona K. 2	8209 ^{ac} ± 1811
Detroit 2 D. R.	8103 ^{ac} ± 1812
Detroit 3	11757 ^{ac} ± 2147
Detroit G.	11788 ^{ac} ± 2186
Formanova	13534 ^{ac} ± 2306
Forono	12177 ^{ac} ± 2179
Gesche SG	12406 ^{ac} ± 2183
Jannis	10737 ^{ac} ± 2031
Jawor	11555 ^{ac} ± 2107
Libero RZ	20489 ^{a} ± 2988
Monty RZ F1	7327 ^{ac} ± 1678
Nobol	12474 ^{ac} ± 2190
Nochowski	4602 ^{cd} ± 1330
Pablo F1	13615 ^{ac} ± 2310
Regulski O.	9359 ^{ac} ± 1896
Robuschka	11129 ^{ac} ± 2067
Ronjana	13002 ^{ac} ± 2236
Hilmar	5190 ^{bcd} ± 1412
Sniezna Kula	9987 ^{ac} ± 1979
Tondo d. Ch.	15947 ^{ad} ± 2678
UB-E3	12745 ^{ac} ± 2235
**(B) Medians based on storage period**	
**Storage period**	
Directly after harvest	9095 ^{B} ± 414
1 month after cold storage	10977 ^{A} ± 459
4 months after cold storage	10997 ^{A} ± 473
